# *‘Am I fixed, am I better now?’*: undergoing MR-guided focused ultrasound for essential tremor: an interpretative phenomenological analysis

**DOI:** 10.3389/fneur.2024.1352581

**Published:** 2024-02-08

**Authors:** Tsvetina Stoycheva, Ayesha Jameel, Peter Bain, Dipankar Nandi, Brynmor Jones, Lesley Honeyfield, Wladyslaw Gedroyc, Jaqualyn Moore

**Affiliations:** ^1^Imperial College Healthcare NHS Trust, London, United Kingdom; ^2^King’s College London, London, England, United Kingdom; ^3^Imperial College London, London, England, United Kingdom

**Keywords:** essential tremor, interpretative phenomenological analysis, MR-guided focused ultrasound, patient experience, patient perspective

## Abstract

**Introduction:**

Essential tremor (ET) is characterised by postural and intentional tremor typically affecting the upper limbs, which can negatively impact functionality and quality of life. Magnetic Resonance-guided Focused Ultrasound (MRgFUS) is a novel and promising non-invasive treatment for ET which offers instantaneous results.

**Methods:**

Using interpretative phenomenological analysis we explored the experience of undergoing MRgFUS in six ET patients as well as their experiences pre- and post-procedure.

**Results:**

One-time, retrospective semi-structured interviews were conducted and six themes emerged: Life pre-treatment: “It’s everyday tasks that get you down” and “Most people who understand, they are okay. Some people aren’t”; MRgFUS: Treatment day: “Going into the unknown” and “There’s no way I was going to press that button”; and Life post-treatment: “One is good. Two is better” and “Am I fixed, am I better now?.”

**Discussion:**

The findings point to a significant period of adjustment associated with living with ET and the effects of undergoing ET MRgFUS treatment. As ET progressed, participants struggled to cope with increasing symptoms and had to develop coping strategies to manage life with ET. The procedure itself was perceived as strange and extraordinary and despite some immediate adverse effects participants were determined to go through with it. Post procedure, all participants reported tremor suppression which was life changing. While some participants still felt burdened by ET, others expressed it took them a while to psychologically adjust to what essentially was their new body. This study has highlighted the need for patients to be supported at all stages of their ET journey.

## 1 Introduction

Essential tremor (ET) is the most common cause of disabling tremor (1) and The National Tremor Foundation estimates it affects approximately 1 million people in the UK. It is characterised by a postural and intentional tremor typically affecting the upper limbs, with some patients also experiencing head, voice and lower limb tremor (2). Tremor is typically symmetrical (3), although the higher amplitude of tremor can vary between the dominant and non-dominant arm (4). ET develops insidiously and progresses slowly over time with tremor generally beginning in the arms and spreading to other body parts in some patients. Apart from the location, the amplitude of tremor in someone with ET can also vary from mild to potentially disabling shaking. Despite an increased prevalence among the elderly, ET can occur at any age. It is thought to have a bimodal age of onset – one peak between the ages of 10 and 20 years, and another between 50 and 60 years (5). ET can be a genetically inherited disorder with approximately 50% of people having a positive family history of the condition (2).

Although “benign” in terms of any possible effects on life expectancy, ET’s clinical characteristics can negatively impact functionality and quality of life (6). Unlike resting tremor (common in Parkinsonism) which occurs when the muscles are relaxed, intentional and postural tremor can affect tasks of daily living such as eating, drinking, dressing and writing. Thus, the diagnosis is associated with significant impairment of manual function, which affects daily activities and results in varying degrees of disability and social handicap (7).

People with ET have been found to be at increased risk of anxiety and depression (5). Chandran et al. (8) suggested that among other factors, depression and anxiety in ET can be attributed to the impact of tremor on every day and work performance with tremor-associated embarrassment leading to low self-esteem and social isolation.

First line of treatment for ET is pharmacological although even well-established treatments can be ineffective in 25–55% of patients and are often associated with serious adverse events in a large percentage of patients (9). Pharmacological agents can lose their efficacy over the course of long-term therapy and in cases where the condition is medically refractory, neurosurgery such as radiofrequency (RF) ablation thalamotomy and deep brain stimulation (DBS) is considered. Although effective, both interventions are invasive procedures and carry significant risks including infection and intracerebral haemorrhage (10).

To mitigate the risks of surgical interventions, MR-guided Focused Ultrasound (MRgFUS) has recently emerged as a novel and promising non-invasive treatment for ET. Focused ultrasound has been used to treat uterine fibroids and prostate cancer in the past, but the recent introduction of phased-array transducers allows incisionless intracranial lesioning under real-time magnetic resonance thermography (11).

In the treatment of ET, the procedure can be performed as a day case and takes approximately 3–4 h to complete. As part of the preparation, the patient’s head is completely shaved, and a stereotactic head frame is attached which aims to eliminate any potential movement during the procedure (12).

Throughout the procedure patients lie supine inside an MRI scanner with their head placed inside a phased-array transducer containing 1,024 elements arranged in a hemisphere. These individual elements are used for beam steering as they focus all ultrasound beams onto a small target to generate heat, which allows thermal ablation of the target brain tissue. To prevent any thermal damage caused by increase in bone temperature, chilled water is constantly circulated around the head (11, 13).

Patients are kept awake throughout the course of the procedure while ultrasound sonications are delivered to ablate the target tissue. An ultrasound sonication lasts 13–24 s on average (11, 13) during which time the patient can press a button to terminate its delivery should they experience any pain or discomfort. Following each sonication the patient is assessed by a neurologist while still lying on the table for any adverse events and tremor suppression. One way of assessing tremor suppression is to ask patients to draw Archimedes’ spirals after each sonication, which visually demonstrate the severity of their tremor and help track response to treatment.

Pilot and sham-controlled studies have focused on the safety and effectiveness of MRgFUS with centres worldwide starting to report data on the procedure’s long-term effectiveness and impact on quality of life. In 2018, The National Institute for Health and Care Excellence (NICE) issued a positive NICE guidance for unilateral MRgFUS in ET. In 2020, NHS England agreed to fund MRgFUS treatment of ET for NHS patients effective from April 2021. A recent health economic study (14) also demonstrated the favourable cost-effectiveness profile of MRgFUS for the treatment of ET in England. With MRgFUS funding in place for ET, it is important to understand patients’ perspectives and experiences of this new type of treatment. Patient-centeredness has been increasingly recognised as a crucial part of quality of care which is sometimes overlooked in the pursuit of treatment efficacy (15). Some evidence suggests that there may be discrepancies between what patients with neurodegenerative conditions and physicians value in terms of the impact of the disease and the focus of treatment (16). While some health professionals may believe that quality of life depends primarily on severity of disease and effectiveness of treatment, patients with neurodegenerative diseases have been found to emphasise other factors including mood (depression) and effective communication with healthcare providers (Janca, 1999, as cited in Findley & Baker, (16)).

In ET, assessing clinical effectiveness and quality of life has often been quantitative in nature. However, considering the complex nature of ET and the novelty of the MRgFUS treatment, qualitative approaches can provide more detailed exploration of patient experience. This article explores the patients’ experience of living with a chronic neurological condition and the impact of MRgFUS on quality of life.

## 2 Methods

An interpretative phenomenological analysis (IPA) approach was used to inform the conceptual background and to guide the study which was conducted between January 2021 and July 2021 at a single centre.

Purposive sampling was adopted with a sample size of 5–15 participants which was in line with an IPA approach. It was anticipated that this sample size would allow gender and age diversity among participants while ensuring sufficient data can be collected to describe in depth the phenomenon under investigation. IPA researchers typically interview small samples, which are fairly homogenous and often chosen through purposive sampling (17). Considering that a small number of interviews is normally sufficient in IPA, the aim is to find a more closely defined group for which the research question will have significance and to understand the perceptions of a particular group rather than make general claims (18).

Potential participants were approached by their healthcare team and those who expressed interest in taking part were interviewed over the phone due to COVID19 restrictions preventing hospital visits. Consent was obtained over the phone prior to all interviews. Adult participants (18 years and above) with confirmed diagnosis of ET who had undergone a unilateral/bilateral MRgFUS procedure were included in the study after consenting to study participation.

Semi-structured interviews were used in line with IPA and an interview guide was developed formulating three neutral, open-ended questions to reflect the three main areas of interest: Living with ET, Undergoing MRgFUS and Life post-procedure. Further prompting questions and conversation continuers were used flexibly to invite participants to give more detail or clarify what was being discussed. The interview guide was initially piloted with appropriate volunteers who were familiar with the MRgFUS procedure. Participants were interviewed once by the first author and the audio-recorded interviews were transcribed before analysis. All interviews are anonymized, and pseudonyms and identifiers have been omitted.

### 2.1 Data analysis

**Figure 1:**
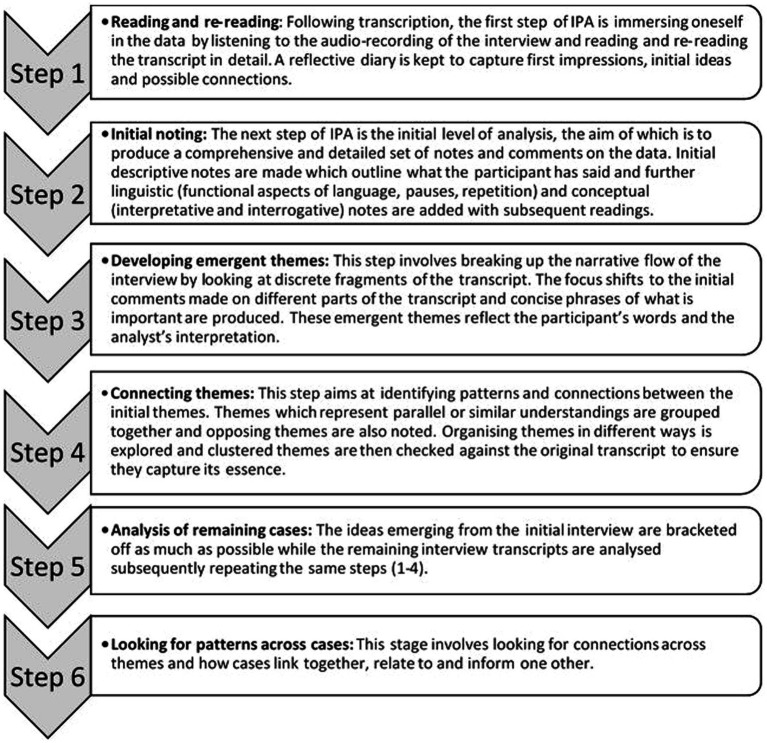
Analysis of interview data: Steps to IPA (19).

In line with IPA, the transcripts were analysed following several steps as defined by Smith et al. (19) (Figure 1): looking for themes in the first case, connecting the themes, continuing the analysis with other cases.

The transcripts were read multiple times, highlighting areas of what was deemed significant discourse. During the process of analytical re-reading notes were made summarising the essence of these excerpts which were then transformed into preliminary themes (phrases best representing what was being said). Through constant comparison, connections were sought between the preliminary themes while referring repeatedly to the transcripts to ensure accuracy. The preliminary themes were then used to form superordinate themes which best capture the essence of patients’ experience.

### 2.2 Ethical considerations

This study received HRA approval and favourable opinion by the London - Westminster Research Ethics Committee (REC reference: 20/LO/0156) in February 2020.

## 3 Findings

### 3.1 Demographic data and clinical outcome

Six patients with ET who had undergone a unilateral or bilateral staged MRgFUS treatment were interviewed. Participants’ age at the time of treatment ranged from 59 to 81 years and the duration of tremor pre-treatment ranged from 7 to 71 years. All participants apart from one were male and all participants but one had undergone a unilateral treatment. According to the participants’ treatment records, the tremor severity pre-procedure varied from mild to severe while post-treatment tremor severity was mild for all participants. On average, interviews took place roughly three and half years post-treatment and lasted around forty-seven minutes. During the interviews participants demonstrated no difficulty in recalling past events, their pre- and post- treatment narratives were detailed and they provided rich accounts of their experience of undergoing MRgFUS.

### 3.2 Themes

The following themes emerged from the six interviews and were organised temporally:

- Life pre-treatment: “It’s everyday tasks that get you down” and “Most people who understand, they are okay. Some people aren’t”
- MRgFUS: Treatment day: “Going into the unknown” and “There’s no way I was going to press that button”
- Life post-treatment: “One is good. Two is better” and “Am I fixed, am I better now?”

#### 3.2.1 Life pre-treatment

##### “It’s everyday tasks that get you down”

All participants gave an account of the physical and emotional challenges of living with essential tremor. Regardless of the time of onset, activities of daily living including “*eating, drinking, baking, carrying drinks, cooking*” were adversely affected with some participants describing the need for assistance with practical tasks from family members. The burden of intention tremor was particularly difficult to manage for all participants:

“*You can’t help but concentrate (…) if you’ve got let’s say a hot drink, because you’re shaking you might spill a bit and you spill it on yourself, that really makes you concentrate and of course then the whole thing goes up in the air literally.”*

Similarly, another participant noted that it was becoming unsafe for him to sometimes do things around the house and when tasks such as DIY work were not completely impossible, they would take disproportionately long to accomplish. Hobbies also “*fell by the wayside*” and employment was negatively affected with some participants taking early retirement as a result. The multifaceted impact of tremor required adaptations to help navigate life with diminishing functional ability. All participants spoke of strategies and coping mechanisms such as “*holding on to one hand or bracing one hand against the side of a table or pushing it up against your body*” that were necessary to manage unilateral hand tremor. Overall, participants experienced increasing frustration in the face of decreasing functionality: “*when you got it 24/7, yeah it’s a different, it’s a different ball game*.” Feelings of annoyance and low mood were often further exacerbated by misdiagnosis or lack of diagnosis over prolonged periods of time following tremor onset. While some participants ultimately “*recognised it as something in the family*,” others were initially struggling to make sense of their ambiguous ill-health experience. The interplay between stress and trembling (typically a physiological sign of stress) was explored to establish a much needed cause-and-effect relationship. For some participants, the strong drive for sense-making served as motivation to gather, attend to and process information in a way which left them questioning the validity of their conclusions:

“*I remember in my teens (…) listening to a radio lecture (…) that (…) parents who’ve had malaria (…) there was a relationship in their children of the virus that lead to very mild forms of tremor. (…) I was quite impressed because I thought* “*hang on, that’s me”. (…) I’ve mentioned this, you know, over the years and nobody’s ever heard of it. Uhm maybe I was dreaming or hypersensitive.”*

This quote demonstrates the emotional burden of a condition with very intrusive symptomatology and the human drive for knowledge and understanding which might lead to a resolution.

##### “Most people who understand, they are okay. Some people aren’t”

Participants described situations in which they would feel embarrassed as a result of the visibility and unpredictability of their tremor. Social occasions such as religious functions, weddings and funerals brought about great levels of anxiety to the point where some participants completely withdrew and restricted their life to “*home to work, work to home, that’s it*.” When it did not result in social withdrawal, the tremor-driven social anxiety led to the development of further strategies to adapt and adjust to the demands of a worsening situation - “*you find ways of eating food without people noticing too much.”* Some participants, however, felt the need to provide an explanation over concerns their tremor might be misinterpreted as something more sinister – “*always used to be frightened that people would think that I was on drugs like, you know, a junkie type*.” While some participants feared the moral judgement associated with society’s perceptions of drug users, others were confronted directly with insensitive questions and remarks including: “*You’re a young person, why are you shaking like this, what’s going on*” and “*you wanna pull yourself together*.” Lack of compassion was sometimes even expressed by healthcare professionals: “*I remember [a doctor] years ago saying ‘Well, I do not think we can be wasting more time so you can do a bit of DIY’*.” The very visible restrictions essential tremor placed upon participants’ social lives similarly affected family members:

*“…my husband likes, likes me to be perfect [laughing] and I remember having a drink in the interval before an opera (…) and I tried to hold the glass and drink with my right hand and it shook and he said* “*Use your left hand” because that was better uhm you know, so it did, it did affect him as well really because he’s a worrier and he likes everything to be just right”*

The idea that tremor was the kind of imperfection that needed sorting, fixing, correcting or repairing was present in all participants’ accounts, carrying a negative undertone and alluding to a sense of guilt.

#### 3.2.2 MRgFUS: treatment day

##### “Going into the unknown”

While participants generally felt confident about undergoing MRgFUS, everyone described to a certain extent experiencing a natural fear of going into an unknown situation:”*…suppose it’s a bit like going to the dentist (…) you do not know what they are going to do until you are in the chair..”* Some participants found themselves feeling uneasy while others had concerns over potential complications during the procedure: “*I was fairly tense wondering what was happening and hoping they’d hit the right spot and were not going to burn my brains out.”*

Participants described that their initial anxiety eased once the pre-operative preparations and set up were completed and they were on the MR table: “*once I been settled down in the MRI machine, it was fine*.” One participant emphasised how once he became familiar with the treatment stages and how the procedure was carried out, it was easier to go through with it:

“*… one of the things is they fetch you out* [of the MR scanner] *and have a chat and, and they put you back in again and [laughing] once (…) you realise uhm that this bit is going to be where they operate and then they would take you out to say* “*How do you feel?” and then push you back in and then you get all the process restarting [pause] you get used to it.”*

The fact that the treatment itself is delivered in sonications and patients are assessed after each one was experienced as helpful and reassuring: “*the amount of times you are sort of wheeled out (…) to see how you are each time (…) that breaks it up into segments which makes it more acceptable*.” Continued communication with the clinical team throughout the treatment played a key role for participants and having family members present on the day also encouraged them to get through the procedure: “*it was excellent for me to have my wife actually in the magnetic room.”*

##### “There’s no way I was going to press that button”

All participants felt that the “*screwing in of the crown*” (fitting of the stereotactic head frame to the skull) was one of the most painful or uncomfortable parts of undergoing the treatment. Once inside the MRI scanner, participants described the treatment as “*strange*” and “*extraordinary*.” There was a sense of rising up to a challenge with some participants describing that they were “*doing what I could do*” and “*it was all sort of questions and people telling me what to do and trying to do it*.” Sensations during the procedure varied among participants with one participant recalling that he “*did not feel a thing*.” Other participants, however, experienced a burning sensation inside the head during sonication delivery which for one participant became so severe that he had to press an emergency button to terminate the energy delivery. Despite the pain and the treatment interruption, he was determined to persevere and did not terminate the procedure. Similarly, another participant described the burning sensation during sonication delivery as “*quite intense (…) I gave it a 7 out of 10,”* however he was also determined to see it through:

*“… the nurse gave me the button, I said* “*I don’t want that, I don’t want that!”. She said* “*Oh, you’ve got to have it” cause there’s no way I was going to press it, no way. I was so on it to get rid of this tremor.”*

Rather than pain, sonication delivery caused two participants to experience a spinning, tumbling sensation: like a “*trapeze artist doing sort of backward somersaults in the air*” which led to dizziness and nausea. Despite this “*sort of disorientation, of tumbling, (…) falling out of control*,” neither of them interrupted a sonication and their treatments were completed. As one participant explained: “*I never thought of stopping, I was determined to see it through.”* Similarly, another participant was so committed to improving his tremor, he felt that going through hardship was worth it: “*mentally once you have built up the confidence, you can write off your pain*.” As one participant suggests: “*I had the tremor bad enough to, to do whatever necessary*.”

Despite these untoward events participants persevered to complete their treatment. The strong drive to go through with the treatment serves to demonstrates the everyday struggle and frustration living with essential tremor brings: “*to be really honest, you get to a position (…) with your tremor that (…) you will have a go with any—most things*.”

#### 3.2.3 Life post-treatment

##### “One is good. Two is better”

All participants experienced immediate improvement of their tremor which was described as “*revolutionary*” and “*brilliant*.” One participant realised his tremor was improving during the treatment when he was asked to repeatedly draw free hand spirals while lying on the MRI table – “*that’s one thing I treasure from both operations (…) my drawings (…) how they got better..”* Similarly, another participant noticed her tremor improving during a different treatment task (pretending to drink from a vitamin bottle): “*it was a sort of pill box with something in it rattling (…) and it rattled less, and then not at all and it was absolutely amazing.”*

Post-procedure side effects from the treatment included fatigue and difficulty with speech and balance which were mild and transient. All participants were adamant that undergoing the treatment and experiencing temporary adverse effects afterwards were worth it considering the tremor reduction they experienced. One participant noticed a big difference post-treatment in small, mundane tasks such as:” *I could put the key in the keyhole straightaway without having to have four goes*” and another recognised the treatment has improved aspects of everyday life including “*eating and drinking, socialising*.” Having one arm treated was very beneficial for participants but those who had bilateral tremor were confined to using their tremor-free side now: “*So I tend to do a lot more things single handed, one handed uhm so the problems arise where I’ve got to use two hands*..” One participant also agreed that tasks and activities which involved using both hands at the same time were still problematic for her: “*still my left hand if I, if I involve it, it can upset something*.” Similarly, another participant struggled with his untreated arm and was disappointed when he was advised not to proceed with a second treatment to address that: “…*I was hoping it could give me that same improvement on the left side which I now cannot really use very much.”*

It appears that while regaining functionality in one arm undoubtedly has a positive impact on day to day life, it also inadvertently draws focus to the lack of functionality of the untreated arm in participants with bilateral tremor.

##### “Am I fixed, am I better now?”

The newly regained functionality in the treated arm also necessitated a period of adjustment for participants. Participants acknowledged they no longer needed their old strategies to cope with the tremor and it took them some time “*to relinquish these habits*” and adjust accordingly which was described as “*a peculiar sensation*”:

*“…the family kept reminding me that I wasn’t left handed - I was right handed, because I was trying to do everything with my left hand. It took—It was a psychological [pause] delay [laughing] in relying on my right hand, maybe two weeks…”*

The striking difference between treated and untreated arm in patients with bilateral tremor had to be processed psychologically.

Participant also needed to let go of the tremor and accept their new level of functionality which was not always easy to do. One participant explained that even now, four years after his treatment he would still “*opt out of volunteering to help serve drinks just in case*.” It seems that the trauma of past tremor-induced embarrassment had a tight grip and it was difficult for participants to let go and feel confident in social situations which they dreaded before. Similarly, another participant also felt that leaving essential tremor behind did not happen automatically but required some time and active effort. Another participant noticed this was particularly driven by the fact that MRgFUS offered instantaneous tremor reduction in the space of hours and the sudden and abrupt disappearance of tremor, while positive and desired, required to be psychologically processed:

“*It’s difficult to put a finger on it but uhm everything you do, you don’t notice these things because they are so gradual uhm and it came to a stop rather suddenly and I thought* “*Oh, that’s not happening and that’s not happening” and uhm the things [pause] you know, you just carry on. I don’t consciously think all the time about uhm* “*Am I fixed, am I better now?” or things like that, you haven’t got time. I mean there’s lots to do in life [laughing]”*

Participants’ narratives suggest that a tremor-free hand does not immediately equate a tremor-free mind and fully letting go of the burden of essential tremor comes after some time.

## 4 Discussion

The findings point to a significant period of adjustment associated with both living with ET and the effects of undergoing MRgFUS treatment for it. As ET progressed, participants struggled to cope with increasing symptoms and the dread and embarrassment tremor brought about in social situations. Participants had to develop coping mechanisms and strategies to manage life with ET and this adjustment period was one of considerable loss of sense of normality. According to Lazarus’s stress and coping theory [e.g., (20)], cognitive and behavioural responses are key elements in the adjustment process. Participants in this study demonstrated behavioural changes in order to physically cope with their tremor, and also employed cognitive adaptations especially in social situations. This was mostly evidenced by their need to provide an explanation for their tremor over fears that it might be associated with addiction. Moore et al. (21) also found that ET patients’ social anxiety was exacerbated by the prospect of misinterpretation of their uncontrollable shaking and unjust moral judgements on their character. It can be argued that this response in ET patients is a cognitive adjustment in an attempt to restore a sense of control and positive self-view in the face of social judgement.

As was evident in participants’ narratives, empirical evidence suggests that living with chronic disease requires adaptations in multiple life domains (22). The restrictions tremor placed on daily practicalities affecting their personal, social and work lives resulted in feelings of annoyance, anxiety, extreme frustration and low mood. A thematic synthesis of the psychological processes of adaptation and hope in MS patients described that the initial cognitive adaptation to multiple sclerosis included similar emotional responses. It also brought feelings of perceived loss of control in life and over disease symptoms and MS patients expressed particularised hopes for improvement or “normality” (23). Similarly, research on the experience of amputation and prosthetic use in adults has identified that initially the key meaning of amputation was a loss of independence and control over life which was sometimes conceived as akin to bereavement (24). The loss of control and independence ET participants reported experiencing also alluded to a sense of loss of normality as often in their narratives they referred to tremor as something that needed to be fixed, sorted or repaired. Similarly, Mathers et al. (25) concluded from their systematic review of PD patients that the feeling of loss takes patients away from a sense of normality which they are trying to regain through treatment. In that sense, participants in the current study, much in keeping with PD patients undergoing DBS (25, 26) did not see MRgFUS as merely an option but rather as an obvious choice.

The procedure itself was perceived as “strange” and “extraordinary” similarly to patients describing DBS as “spectacular” and “mysterious” (26). Participants reported that one of the most uncomfortable parts of the procedure was the placement of the stereotactic head frame. This is somewhat in keeping with Ben-Haim and Falowski’s (27) survey of DBS patients who reported an average comfort level with head frame placement of 5.2 (+/− 3.15) out of 10, with 10 representing “very uncomfortable.” Nonetheless, being observed by family and clinicians throughout the treatment was experienced as reassuring and helpful. This highlights the importance of continuous and effective communication between treating team and patient but also the value of having family present in the treating room. Indeed, patient-clinician communication has been highlighted as a significant factor in patient satisfaction and complaints about care (28) and it plays a key role in healthcare service quality (29).

Despite some participants experiencing adverse effects during the treatment, all participants’ narratives demonstrated an incredible sense of determination and perseverance throughout the treatment process which can be interpreted as stemming from a life of discomfort and frustration caused by ET.

Post procedure, all participants reported tremor suppression which was life changing. This was very similar to PD patients describing DBS as a “miracle,” “life-changing” and “unbelievably wonderful” (30, 31). Both participants in the current study and in the existing DBS literature expressed that the most significant impact of their respective treatments was evident in everyday achievements such as eating, drinking and socialising. While for DBS patients this allowed the return of a much desired sense of normality, for some of the MRgFUS participants in the current study it inadvertently drew attention to the unilaterality of their procedure. The likely explanation for this is that DBS can be performed bilaterally during the same operation if a patient has troublesome symptoms on both sides of their body. MRgFUS, on the other hand, can so far only be performed as a staged bilateral treatment with two separate procedures taking place at least 9 months apart due to concerns over speech adverse effects.

In the current study, the fact that the treated arm brought focus to the untreated one anecdotally resembles patients’ initial experience of prosthesis use. For instance, Murray et al. (24) found that introducing prosthesis was often emotionally ambiguous for patients who appreciated the undoubted benefit of added functionality an artificial limb can afford but continued to feel the profound loss of their limb. Perhaps for ET patients, while a one-sided MRgFUS treatment brings back a much desired functionality, they continue to experience a sense of loss of that same functionality on the contralateral side.

Participants overwhelmingly expressed that undergoing the treatment necessitated an unexpected period of adjustment. This is entirely in keeping with previous research on DBS patients who reported difficulty breaking old habits and getting used to a new level of functionality (25, 30). Another form of adaptation which was required post-MRgFUS related to letting go of the tremor altogether and of past negative experiences caused by it. Some participants in the current study still seemed haunted by the burden of ET while others expressed it took them a while to psychologically adjust to what essentially was their new body. In the amputation and prosthesis use literature, the term “embodiment” (the perception of the prosthesis as part of one’s body) is often an important component of functional and emotional recovery (32). In an IPA study of the embodiment of artificial limbs, Murray (33) identified time as a crucial component of the adjustment process as with time the use of the prosthesis can become intuitive and more natural. While in ET, regaining functionality is not associated with some of the negatives reported with the use of artificial limbs such as people staring (34), time can still be an important factor in the adaptation process. Indeed, Mathers et al.’s (25) idea that a cured body may not necessarily equate a cured mind post DBS seems to apply to MRgFUS patients alike, signifying a crucial period of adjustment following interventions capable of drastically reducing or completely abolishing very troublesome symptomatology instantaneously.

### 4.1 Conclusions and limitations

This study has highlighted the need for patients to be supported at all stages of their ET journey by linking them to appropriate resources and existing networks (such as The National Tremor Foundation, The Focused Ultrasound Foundation), creating dedicated support groups and also by easing the adjustment to the very sudden reduction of tremor post-procedure which appears to be both psychological and physiological in nature.

One important consideration for the MRgFUS service outside of the treatment of ET is the sheer determination to be treated ET patients demonstrate during this awake procedure. Due to the chronic nature of ET, these patients may well be more likely to persevere during the MRgFUS procedure, and different patient populations need to be carefully considered for treatment before assuming they will share this similarity. For instance, for applications of MRgFUS in brain tumour work, factors such as patients’ psychological and physical adjustment to their diagnosis, prognosis and previous treatments need to be taken into account. Similarly, in PD the “off state” patients experience when they are not taking PD medication needs to be accounted for since it is likely patients would not be on their typical medical management during MRgFUS for the effects of the treatment to be accurately assessed.

The scope of this study was limited by University course requirements and the length of time between the interviews taking place and participants’ respective procedures which may have resulted in poorer recall of very specific details and some negative aspects of the procedure being overlooked.

Future research should explore patient experience of MRgFUS as part of the NHS service, including other patient groups and interviewing patients before and after their treatment rather than collecting a retrospective account of all events.

## Data availability statement

The datasets presented in this article are not readily available because data are not available due to confidentiality. Requests to access the datasets should be directed to tina.stoycheva@nhs.net.

## Ethics statement

The studies involving humans were approved by London - Westminster Research Ethics Committee. The studies were conducted in accordance with the local legislation and institutional requirements. Written informed consent for participation was not required from the participants or the participants’ legal guardians/next of kin because Consent was obtained over the phone prior to any study related activity due to COVID19 restrictions preventing hospital visits. This was agreed with the REC.

## Author contributions

TS: Conceptualization, Data curation, Formal Analysis, Methodology, Project administration, Writing – original draft, Writing – review & editing. AJ: Writing – review & editing. PB: Conceptualization, Writing – review & editing. DN: Writing – review & editing. BJ: Writing – review & editing. LH: Writing – review & editing. WG: Supervision, Writing – review & editing. JM: Methodology, Supervision, Writing – review & editing.
